# Empowering workforces in AI-driven environments: co-skilling, organizational support, and mitigating job insecurity

**DOI:** 10.3389/fpsyg.2025.1700129

**Published:** 2025-12-19

**Authors:** Yujie Zhang, Xiaoxiao Liu, Qiao Yan, Meng Na

**Affiliations:** 1 School of Business, Zhuhai College of Science and Technology, Zhuhai, Guangdong, China; 2 School of Management, Guangdong University of Science and Technology, Dongguan, Guangdong, China; 3 Graduate School of Business, Universiti Kebangsaan Malaysia (UKM), Bangi, Selangor, Malaysia

**Keywords:** artificial intelligence, co-skilling, employee readiness for AI, job insecurity, mental wellbeing, perceived organizational support, skill confidence

## Abstract

The rise of artificial intelligence (AI) has transformed workplaces, creating opportunities for innovation but also heightening job insecurity (JIN) among employees. This study examines the impact of Co-Skilling dimensions—participation, engagement, peer collaboration, and learning effectiveness (LE)—on mitigating JIN through perceived organizational support (POS), mental wellbeing (MEW), and skill confidence (SC). Using the Social Learning Theory (SLR) and the JD-R model as theoretical underpinnings, the research highlights the moderating role of employee readiness (ER) for AI in shaping these dynamics. A cross-sectional quantitative design with stratified random sampling was employed, involving 437 responses from employees across manufacturing, healthcare, technology, banking, and retail industries in China. Results demonstrate the significant mediating effects of POS, MEW, and SC, with POS emerging as a critical buffer against insecurity. However, nonsignificant findings in certain SC-related pathways and moderation effects of ER underscore the complexity of addressing JIN in technologically dynamic environments. This study contributes to theory by expanding the applications of SLR and the JD-R model and offers practical implications for organizations to design tailored Co-Skilling initiatives. Future research should explore additional contextual and psychological factors to enhance workforce adaptability in AI-integrated workplaces.

## 1 Introduction

The integration of artificial intelligence (AI) and robotics into workplaces has triggered profound transformations across industries, reshaping job demands and employee roles. Recent estimates from the World Economic Forum project that 85 million jobs could be displaced in 2025 due to AI and automation, even as 97 million new roles are created, underscoring both the disruptive and generative potential of these technologies (Russo, 2020). While AI promises enhanced efficiency and innovation, its widespread adoption has also fueled significant job insecurity (JIN), particularly among employees performing routine and automatable tasks (Masayuki, 2017; Yam et al., 2023). This dual-edged nature of AI presents a critical challenge for organizations aiming to balance technological advancement with workforce stability. As research indicates, roles emphasizing personal interaction and soft skills are less vulnerable to automation, reinforcing the need for employees to develop competencies that complement AI technologies (Coupe, 2019; Hussain, 2024). For instance, in the financial sector, AI-powered chatbots now handle up to 60–80% of routine customer inquiries, shifting employees toward higher-level analytical and client-facing responsibilities (Roman, 2024).

Despite ongoing discussions on upskilling and reskilling to meet these new demands, limited exploration exists regarding collaborative Co-Skilling initiatives—programs that emphasize shared learning, reciprocal knowledge transfer, and teamwork (Al-Asfour and Zhao, 2024; Cramarenco et al., 2023). While large organizations such as Amazon have announced multi-million-dollar investments to retrain employees for more advanced roles, much of the focus remains on individual upskilling rather than collective efforts that leverage team-based learning. Although studies acknowledge the importance of continuous skill development in AI-driven environments, they seldom examine the specific processes through which employees collectively acquire and refine capabilities that mitigate fear of automation.

JIN, a multifaceted stressor subjectively perceived by workers, has long been studied for its pervasive effects on employee wellbeing, engagement, and organizational commitment (De Witte, 2005; Sverke and Hellgren, 2002). In the context of AI adoption, employees often view automation as a direct threat to job stability, leading to heightened stress, disengagement, and turnover intentions (Jaiswal et al., 2021; Thakkar et al., 2020). Although measures such as job security guarantees can temper these concerns (Bryson et al., 2009), more recent findings point to the role of perceived organizational support (POS) in shaping employees’ sense of safety amid AI-induced disruptions (Hou and Fan, 2024; Li, 2023; Oglesby et al., 2024). Still, research has yet to explore fully how organizations can deploy Co-Skilling strategies to bolster POS and assuage fears arising from technological displacement. Notably, an IBM Global AI Adoption Index (2022) revealed that 45% of global executives cite “workforce readiness” as a key barrier to successful AI integration, underscoring the pressing need for supportive, collaborative programs.

In parallel, mental wellbeing (MEW) emerges as a potentially vital mediator in the link between AI adoption and employee outcomes, yet its precise role in mitigating JIN remains underexamined (Aulia and Lin, 2024; Chen et al., 2024). Furthermore, discussions of skill confidence (SC)—distinct from general psychological capital—are notably scarce, leaving open questions about its contribution to fostering employees’ adaptability and resilience in the face of automation (Hou and Fan, 2024; Nabawanuka and Ekmekcioglu, 2024). At the same time, employee readiness (ER) for AI is often mentioned in terms of trust or proactive personality (Kong et al., 2024), but few studies have investigated how readiness moderates the effectiveness of POS, MEW, and SC in reducing perceived threats.

Against this backdrop, the present study investigates how Co-Skilling dimensions (participation and engagement, peer collaboration, and learning effectiveness) interact with POS, MEW, and SC to influence JIN in AI-enabled workplaces. By situating the investigation within Social Learning Theory (SLR) (Bandura, 1977; Emilly and Sudi, 2024) and the JD-R Model (Bakker and Demerouti, 2014), this research offers a multifaceted framework that accounts for the interplay of psychological, organizational, and individual readiness factors. In doing so, it addresses existing gaps by highlighting collaborative skill-building processes and examining the mediating and moderating mechanisms that shape employee adaptation in the era of AI.

The findings of this study hold both theoretical and practical implications. Theoretically, the research contributes to the expansion of SLR and the JD-R Model in technologically dynamic contexts, shedding light on the interplay between learning, psychological resources, and JIN. Practically, it provides actionable strategies for designing Co-Skilling initiatives that not only address technological disruptions but also enhance employee wellbeing and engagement. By focusing on the psychological and organizational dimensions of workforce adaptation, this study seeks to advance the discourse on navigating the evolving job market in the age of AI.

## 2 Literature review

### 2.1 Co-skilling

The concept of Co-Skilling, encompassing participation (Mendoza-Chan and Pee, 2024), engagement (Dăniloaia and Turturean, 2024), peer collaboration (Pee, 2022), and learning effectiveness (LE) (Pee, 2020), represents an essential strategy for organizations navigating rapidly evolving technological and workplace landscapes. Co-Skilling initiatives are particularly significant in AI-driven environments, where they equip employees to adapt, reduce job insecurity (JIN), and ensure long-term employability. As Stephany (2020) notes, cross-skilling enhances economic resilience by aligning existing competencies with evolving technological demands, thereby increasing workforce flexibility. Partnerships between academic institutions and industries—particularly in STEM and digital fields—foster applied problem-solving and technological readiness (Pee, 2022), while integration of formal, non-formal, and informal learning approaches remains critical for cultivating an AI-ready workforce (Misko, 2008).

Recent studies underscore the strategic importance of reskilling and upskilling in sustaining organizational agility. Muchiri (2022) emphasizes that these investments are not merely competitive advantages but essential responses to global market dynamics. In manufacturing, the introduction of collaborative robots illustrates this necessity, as workers must undergo reskilling to adapt to automation (Dornelles et al., 2023). The growing literature on AI-era workforce development reinforces these insights: Lai et al. (2025) identify a 23% annual growth in global research on reskilling and upskilling since 2022, while Tambe (2025) demonstrates that algorithmic literacy among non-technical employees substantially improves organizational returns on AI investment. Similarly, Li et al. (2025) provide empirical evidence that workforce adaptability and continuous learning significantly enhance organizational sustainability in technology-intensive contexts. These findings collectively affirm that Co-Skilling—by embedding learning within collaborative and iterative processes—is central to building both individual and organizational resilience.

The dimensions of Co-Skilling—participation (PE), peer collaboration (PC), and learning effectiveness (LE)—operate as interconnected mechanisms within dynamic learning ecosystems. Participation and collaboration foster negotiation and mutual enrichment processes vital for knowledge sharing in workplace settings (Impedovo, 2021). In digital contexts, online co-creation and peer engagement enhance cognitive and epistemic learning, enabling employees to manage complex AI-related challenges (Pee, 2020; Willis et al., 2012). For instance, Carvalho and Santos (2022) found that technology-enhanced peer-learning programs increase metacognitive awareness and collaboration—skills crucial for problem-solving in diverse teams.

From a workplace-learning perspective, Co-Skilling leverages participatory practices to promote engagement and active learning. Billett (2001, 2002, 2004) highlights the dual importance of workplace affordances and individual agency in fostering effective learning. Structured Co-Skilling activities, such as peer-led simulations or collaborative coding exercises, develop technical proficiency while enhancing interpersonal and metacognitive skills essential for teamwork. Xu et al. (2025) further illustrate how AI-enabled production systems in the construction sector require continuous learning cycles that combine technical mastery with collaboration across digital platforms.

Despite its growing relevance, most Co-Skilling research remains heavily quantitative and employee-centric, focusing primarily on individual perceptions. Future inquiry should adopt qualitative and multi-stakeholder designs that include employees, supervisors, and HR leaders to understand how co-skilling practices are conceptualized, implemented, and experienced across organizational hierarchies. Such approaches can reveal contextual nuances—how managerial expectations, resource allocation, and peer dynamics jointly shape engagement and learning effectiveness (Oliveira et al., 2025). Multi-actor inquiry would thus deepen understanding of how organizational intent and individual agency intersect to sustain Co-Skilling ecosystems.

Existing studies also tend to examine Co-Skilling within single-country contexts, limiting understanding of institutional and cultural contingencies. Comparative research is needed to determine whether the relationships among participation, collaboration, and learning effectiveness vary across collectivist and individualist cultures or advanced and emerging economies. Regional evidence supports this view: Ahmed et al. (2025) show that Malaysian employees’ adaptability to AI depends strongly on culturally embedded learning orientations and institutional support for lifelong learning. Such cross-country analyses could clarify how national policy frameworks, labor norms, and digital infrastructures mediate organizational capacity for AI-driven Co-Skilling.

Beyond skill acquisition, Co-Skilling fosters belonging and professional identity, especially in diverse and hybrid workplaces. Peer collaboration and shared goals enhance identification with both the profession and the organization (Willis et al., 2012). Digital collaboration tools further amplify these effects by connecting geographically dispersed teams, improving coordination, and strengthening interpersonal cohesion (Liu et al., 2020). In AI-mediated contexts, Co-Skilling thus serves as a bridge between individual competencies and collective objectives, embedding adaptability and resilience within the organizational culture.

Integrating Co-Skilling into organizational strategies constitutes a proactive measure to empower employees in addressing complex technological challenges. By emphasizing participation, collaboration, and continuous learning, organizations cultivate supportive environments that enhance adaptability and mitigate the uncertainties of AI transformation. As highlighted in recent management analyses (BCG, (Boston Consulting Group), 2025), firms that prioritize human-centered Co-Skilling alongside technological investment are better positioned to achieve sustainable, innovation-driven growth. Consequently, a deeper cross-country and multi-stakeholder exploration of Co-Skilling is imperative for understanding how these initiatives mediate and mitigate the workforce disruptions of the AI era.

### 2.2 Theoretical underpinning

Social learning theory (Bandura, 1977) underscores the role of observation, modeling, and reinforcement in learning, making it particularly relevant in understanding how employees acquire and refine skills through Co-Skilling. In the context of AI-enabled workplaces, the theory emphasizes that learning occurs in social and collaborative settings, where individuals learn not only from their own experiences but also by observing and interacting with peers. This aligns with findings that occupational self-efficacy mediates the relationship between JIN and work-related learning, with temporary workers often engaging in upskilling to enhance employability (Rajthilak et al., 2021; Van Hootegem et al., 2021).

Co-Skilling programs, as conceptualized in this study, embody the principles of expansive learning, a concept that addresses the challenges of collaborative problem-solving and continuous skill development in dynamic work environments (Engeström, 2007). For instance, collaborative AI training workshops allow employees to model desired behaviors, share strategies for coping with workplace changes, and receive feedback, reinforcing their self-efficacy and adaptability. Research on career decision-making further substantiates the role of learning experiences and environmental conditions in shaping employees’ readiness for change and skill acquisition (Mohd Yunus et al., 2024).

Moreover, SLR has been extended to various workplace behaviors, such as self-management and attendance (Frayne and Latham, 1987), highlighting its relevance for understanding employee engagement in structured Co-Skilling initiatives. By emphasizing reinforcement mechanisms, such as recognition and support from organizational leaders, the theory explains how Co-Skilling fosters not only skill development but also positive organizational attitudes and reduced JIN. These insights align with findings that individuals who perceive strong peer and organizational support are more likely to engage in proactive learning behaviors, enhancing their capacity to navigate technological transitions (Emilly and Sudi, 2024; Velayudhan and Palanisamy, 2017).

The JD-R Model (Bakker and Demerouti, 2017; Demerouti et al., 2001) offers a complementary perspective by explaining how job demands (e.g., AI-driven transitions) and job resources (e.g., Co-Skilling, POS) interact to shape employee wellbeing, motivation, and performance. The model posits that job resources can buffer the adverse effects of job demands, a principle that resonates with the role of Co-Skilling initiatives in mitigating the stress associated with AI-driven JIN.

This study identifies Co-Skilling as a critical job resource, reinforcing findings that such resources promote engagement, reduce burnout, and foster organizational commitment (Bakker et al., 2010; Schaufeli and Taris, 2013). For example, structured Co-Skilling activities that focus on AI skill-building enhance employees’ sense of mastery and psychological safety, directly addressing the stressors linked to technological disruptions. Furthermore, the JD-R model’s emphasis on individual strategies, such as coping and self-regulation (Demerouti, 2018), aligns with this study’s findings on the mediating roles of MEW and SC in reducing JIN.

**Figure 1:**
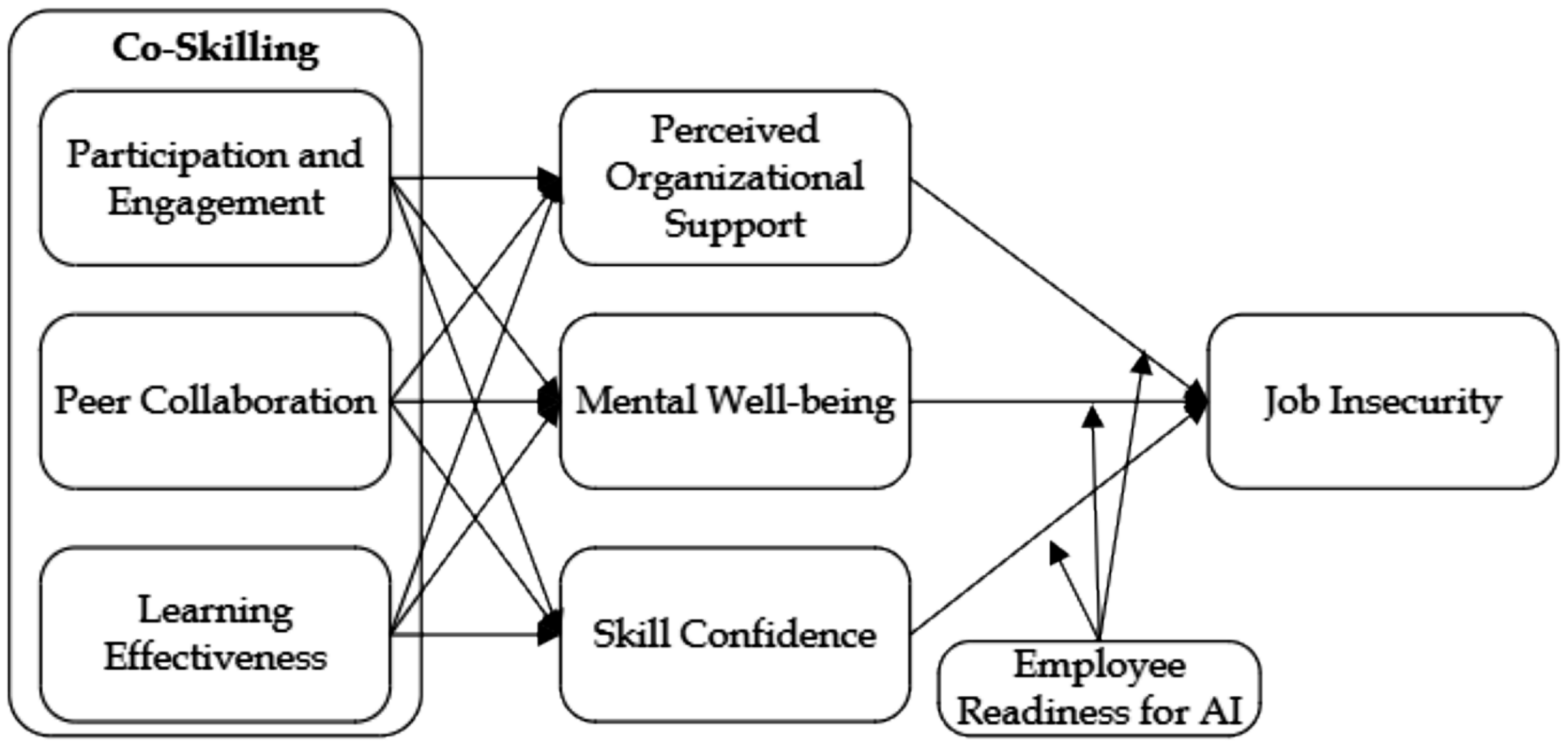
Research framework.

The JD-R model also integrates psychological contract fulfillment as a mechanism linking job resources to organizational outcomes (Birtch et al., 2015). Co-Skilling initiatives fulfill this psychological contract by visibly demonstrating the organization’s commitment to employee development, thereby enhancing trust, loyalty, and engagement. This aligns with findings that job resources, such as POS and SC, not only buffer job demands but also foster proactive coping behaviors and sustained motivation in AI-integrated workplaces (Potocka and Waszkowska, 2013; Sun et al., 2010) (Figure 1).

By synthesizing SLR and the JD-R Model, this study provides a robust theoretical foundation for understanding the dynamics of Co-Skilling in AI-enabled environments. SLR elucidates the mechanisms of observational and collaborative learning, while the JD-R Model contextualizes these processes within the broader framework of job demands and resources. Together, these theories explain how Co-Skilling initiatives enhance psychological resources (POS, MEW, SC) and mitigate JIN by addressing both individual and organizational needs.

### 2.3 Hypothesis development

#### 2.3.1 Co-skilling dimensions and immediate outcomes

POS defined as employees’ perception of their organization’s care for their wellbeing and recognition of their contributions (Sindu and Sujatha, 2024), POS significantly impacts engagement, satisfaction, and psychological health (Alkasim and Prahara, 2020; Eisenberger and Stinglhamber, 2011). It also fosters key psychological states, such as confidence and resilience, which are vital in dynamic work environments (Diana and Frianto, 2021). Organizational initiatives that actively demonstrate investment in employee development (Dachner et al., 2021), such as Co-Skilling programs, are strongly aligned with the mechanisms that enhance POS (Imran et al., 2020).

Co-Skilling participation and engagement are integral to creating a sense of support within the organization (Whelan and Carcary, 2011). Participation in collaborative skill-building activities fosters peer-to-peer interaction and underscores the organization’s commitment to employee growth (Terekhin and Aurora, 2024). For example, when employees in a retail organization collectively engage in data analytics training, the collaborative environment and organizational sponsorship reinforce perceptions of being valued. Research supports this, showing that collaborative activities can reduce workplace stress, strengthen social bonds, and provide employees with a sense of accomplishment (Dunne et al., 2021; Perkins et al., 2020).

Beyond POS, such collaborative environments contribute to employees’ MEW by promoting emotional stability and reducing workplace anxiety (da Reis Silva, 2024). Studies in educational and workplace contexts suggest that active participation in structured learning environments helps individuals regulate emotions, enhance their sense of purpose, and build psychological resilience (Rehman et al., 2023; Russell et al., 2023). These effects are critical in mitigating the mental toll associated with rapidly evolving organizational demands and technological disruptions.

Co-Skilling programs also play a crucial role in fostering SC (Van Tam, 2024). As the belief in one’s ability to acquire and effectively apply new skills, SC is essential for workplace adaptability and performance (Mahedi Hasan et al., 2024). Evidence shows that employees who participate in collaborative learning experiences, such as group problem-solving or technical workshops, exhibit increased self-efficacy and readiness to tackle complex challenges (Billett, 2001; Maurer, 2001). For instance, IT teams engaging in Co-Skilling to learn new software tools reported greater confidence in addressing technological transitions, facilitating smoother organizational adaptation to innovation (Day et al., 2007). Thus, based on the above arguments and evidence, the following hypothesis is proposed:

*H1*: Co-Skilling participation and engagement positively influence (a) perceived organizational support, (b) mental wellbeing, and (c) skill confidence.

#### 2.3.2 Co-skilling peer collaboration

POS is influenced by both organizational practices and interpersonal interactions, with coworker support playing a critical role in shaping employees’ perceptions of organizational care and fairness (Hayton et al., 2011; Simosi, 2012; Stinglhamber et al., 2020). Employees who perceive robust support from their peers and the organization reciprocate with enhanced commitment, loyalty, and productivity (Hakeem and Nisa, 2016). Co-Skilling peer collaboration, as a structured mechanism for fostering coworker interaction and teamwork, directly contributes to these outcomes by cultivating trust and shared purpose (Cascade Team, 2023). For instance, an engineering team collaborating on design improvements is not only likely to generate innovative solutions but also to build stronger interpersonal bonds, reinforcing their perception that the organization values their contributions.

The role of collaborative environments in improving MEW is well-documented across various contexts. In healthcare, peer support groups have demonstrated the ability to reduce isolation, enhance coping strategies, and foster resilience (Johnson and Riley, 2021; Kane et al., 2023). Similarly, in workplace settings, collaboration among customer service teams sharing strategies to manage challenging clients has been shown to alleviate stress and boost collective morale. Research on skill development for underserved women further supports the argument that collaborative learning enhances both practical skills and psychological health by building networks of mutual encouragement and support (Rehman et al., 2023). Programs like peer-supported smoking cessation initiatives highlight the dual advantage of peer collaboration in improving mental health and building confidence (Ashton et al., 2012).

Peer collaboration also plays a crucial role in fostering SC by creating opportunities for shared learning and feedback. In nursing education, for example, students participating in peer-to-peer simulations for mastering life-saving techniques report improved self-confidence and satisfaction (Curtis et al., 2016). Similarly, in corporate environments, peer mentorship programs—such as pairing junior and senior software developers—accelerate skill acquisition and confidence building for novices. Collaborative approaches are particularly impactful for individuals with initially low self-efficacy, as they provide supportive environments for iterative learning and skill refinement (Markowski et al., 2021; Qiu and Lee, 2020).

In the context of Co-Skilling, peer collaboration enables employees to actively engage with their colleagues, fostering a culture of mutual respect and shared responsibility (Nguyen, 2023). These interactions strengthen employees’ belief in their ability to acquire and apply new skills while reducing the psychological barriers associated with workplace challenges (Baker et al., 2021). For example, a retail team collectively undergoing sales training may find that shared experiences enhance their confidence in applying customer engagement techniques. This dynamic not only improves individual capabilities but also reinforces the perception of organizational investment in their development.

*H2*: Co-Skilling peer collaboration positively influences (a) perceived organizational support, (b) mental wellbeing, and (c) skill confidence.

#### 2.3.3 Co-skilling learning effectiveness

LE is a critical determinant of organizational success (Leonard and Marquardt, 2010), particularly in the context of Co-Skilling initiatives that aim to enhance workforce adaptability (Liu et al., 2020). POS is a key factor linking LE to broader organizational outcomes (Musenze et al., 2022). POS reinforces employees’ sense of being valued and supported, thereby motivating engagement in skill-building activities and fostering loyalty (Ahmad et al., 2014; Zumrah et al., 2012). High-quality learning programs—such as leadership workshops or technical training sessions—serve as visible demonstrations of an organization’s commitment to employee development, further amplifying POS (Lancaster and Di Milia, 2014). For example, a manufacturing company offering AI-focused training programs not only equips employees with new competencies but also strengthens their perception of the organization as a supportive partner in their career growth.

Co-Skilling LE is characterized by structured, collaborative activities that foster skill acquisition and knowledge sharing (Pee, 2020). These experiences create meaningful connections between employees and their organizational goals, reinforcing the perception of organizational care. Research on mentoring relationships, where structured guidance benefits both mentors and mentees, illustrates how effective learning environments bolster POS while simultaneously driving professional growth (Bear, 2018).

LE also has a significant impact on MEW (Guoyan et al., 2023). Participating in targeted skill-building programs helps individuals develop resilience and reduce workplace stress by instilling a sense of accomplishment and purpose (Watson et al., 2018). For instance, disadvantaged workers participating in upskilling programs often report improvements in both their skillsets and psychological health, highlighting the dual benefits of learning interventions (Briand et al., 2023; Hammond, 2004a; Hammond, 2004b). However, poorly designed programs or excessive skill demands may lead to frustration and anxiety, underscoring the importance of balancing learning objectives to maximize benefits (Zhu and Chen, 2016).

In addition, Co-Skilling enhances SC, which refers to employees’ belief in their ability to acquire and apply new skills effectively (Mendoza-Chan and Pee, 2024). Confidence plays a pivotal role in workplace adaptability and success (Shabeer et al., 2023). Collaborative learning environments, such as team-based leadership programs or hands-on workshops, empower participants to tackle challenges with greater self-assurance. For example, leadership programs that involve role-playing conflict resolution scenarios have been shown to increase participants’ belief in their decision-making abilities (Leonard and Marquardt, 2010). Moreover, collaborative training approaches particularly benefit employees with low initial confidence by providing supportive structures for incremental skill development (Jones and Good, 2016; Norman and Hyland, 2003).

By embedding skill-building into team-oriented, supportive frameworks, Co-Skilling fosters not only practical expertise but also the psychological resources needed to navigate complex work environments. For example, employees in the technology sector engaging in group-based coding challenges may find their confidence in software application grows in tandem with their technical knowledge, equipping them to address future challenges with resilience and competence.

Thus, based on the above arguments, the following hypothesis is proposed:

*H3*: Co-Skilling learning effectiveness positively influences (a) perceived organizational support, (b) mental wellbeing, and (c) skill confidence.

#### 2.3.4 Perceived organizational support (POS) as a mediator

Extant research highlights that POS mediates the relationship between JIN and various positive outcomes, including organizational citizenship behavior, work engagement, and employee performance (dos Santos et al., 2022; Vera Andriyanti and Suardana, 2023; Yuni and Wahyu Pratiwi, 2020). This mediation effect stems from the ability of POS to signal the organization’s commitment to employees’ wellbeing, which alleviates the psychological burden of insecurity and fosters resilience (Di Stefano et al., 2020; Haar and Brougham, 2011).

POS is significantly influenced by organizational practices that emphasize employee development (Eisenberger et al., 2020). Co-Skilling dimensions—namely participation and engagement, peer collaboration, and LE—foster POS by demonstrating the organization’s investment in its workforce (Pee, 2020). For example, a technology company offering team-based Co-Skilling workshops focused on AI upskilling sends a clear message that it values employee growth, thereby reinforcing trust and loyalty. This aligns with evidence from workplace training programs showing that aligning employee development with strategic goals strengthens employees’ perceptions of organizational support (Bear, 2018; Lancaster and Di Milia, 2014).

The buffering role of POS is particularly critical in mitigating the effects of JIN. Employees who perceive high levels of organizational support report reduced stress, greater engagement, and stronger psychological empowerment, even during periods of significant organizational change (dos Santos et al., 2022; Getahun Asfaw and Chang, 2019). For instance, in the context of a healthcare organization implementing AI automation, employees who experienced clear communication and skill development opportunities were less likely to feel insecure about their roles. These findings underscore the role of POS in enabling employees to adapt to evolving work environments while maintaining their sense of security and commitment (Ahmad et al., 2014; Yulia and Sanusi, 2021).

Co-Skilling initiatives directly contribute to POS by fostering collaborative and goal-oriented learning environments. For example, a retail team collaboratively brainstorming innovative merchandising strategies in a Co-Skilling session is likely to perceive the organization’s support as a driving force behind their collective success. These initiatives not only enhance individual competencies but also strengthen employees’ trust in their organization’s intentions, creating a positive feedback loop between development opportunities and perceived support (Homklin et al., 2014; Simosi, 2012).

Given these considerations, the relationship between Co-Skilling dimensions and JIN is mediated by POS, which acts as a buffer against the psychological challenges posed by uncertainty in the workplace. By signaling organizational investment in employee development, Co-Skilling reinforces trust and resilience, reducing the perceived threats associated with job instability.

*H4*: Perceived organizational support mediates the relationship between (a) Co-Skilling participation and engagement, (b) Co-Skilling peer collaboration, (c) Co-Skilling learning effectiveness and job insecurity.

#### 2.3.5 Mental wellbeing as a mediator

Job insecurity often manifests in heightened stress, anxiety, and reduced life satisfaction, impairing employees’ psychological safety (Aldawsari and Mabkhot, 2023; Sulaiman et al., 2020). This psychological strain undermines engagement, productivity, and long-term wellbeing. To address these challenges, researchers emphasize interventions that foster resilience and emotional regulation through structured support systems (Menéndez-Espina et al., 2019; Sulaiman et al., 2020).

Mental wellbeing, characterized by emotional stability and psychological health, plays a pivotal mediating role between workplace factors and JIN. Employees with high MEW exhibit stronger resilience to workplace stressors, enabling better engagement and performance (Haar and Brougham, 2011; Kundi et al., 2020). For instance, teachers in high-stress environments showed improved performance and engagement when their MEW was supported by peer networks and access to mental health resources (Van Hootegem et al., 2021).

Co-Skilling initiatives, such as collaborative workshops and cross-functional brainstorming sessions, directly enhance MEW by fostering social connectedness and structured development. For example, employees participating in peer-led coaching programs reported reduced anxiety and improved emotional regulation, illustrating the psychological safety generated through shared learning environments (Briand et al., 2023; Hammond, 2004a; Hammond, 2004b). Additionally, mental health-focused skill-building programs, such as Recovery Colleges, empower participants in high-stress settings, reducing emotional turmoil and fostering purpose (Field, 2009; Rehman et al., 2023).

For instance, employees engaging in team-based Co-Skilling activities, like hackathons or innovation sprints, experience collective emotional reinforcement. These settings foster psychological safety by allowing employees to navigate challenges collaboratively, thereby mitigating the negative effects of JIN (Day et al., 2007; Hammond, 2004a; Hammond, 2004b). MEW, as a mediator, highlights the transformative impact of such environments in reducing employees’ perceived threats to their job security.

*H5*: Mental wellbeing mediates the relationship between (a) Co-Skilling participation and engagement, (b) Co-Skilling peer collaboration, (c) Co-Skilling learning effectiveness and job insecurity.

#### 2.3.6 Skill confidence as a mediator

Job insecurity directly impacts employees’ confidence in their ability to acquire and apply new skills, often leading to diminished participation in skill-building and lower performance outcomes (Van Hootegem et al., 2023; Van Hootegem and De Witte, 2019). Conversely, it can also motivate employees to proactively seek training opportunities to enhance employability, creating a dual effect on development behaviors (Van Hootegem and De Witte, 2019).

Skill confidence, defined as self-efficacy in acquiring and applying competencies, mediates the relationship between workplace initiatives and JIN. For instance, employees with high SC perceive learning opportunities as tools for advancement rather than responses to job threats, leading to greater engagement in upskilling programs (Kuvalekar and Lipnowski, 2020). This dynamic highlight the crucial role of SC in transforming JIN into proactive development behavior.

Co-Skilling initiatives, such as peer-led simulations in healthcare or collaborative coding projects in technology teams, significantly enhance SC. These environments provide validation through constructive feedback and role modeling, enabling employees to build self-efficacy incrementally. For example, pairing junior software engineers with experienced mentors in team projects accelerates skill acquisition and bolsters confidence in applying technical solutions (Norman and Hyland, 2003). Similarly, collaborative sales training programs, where participants shadow senior colleagues, have shown to improve both performance and confidence outcomes (Markowski et al., 2021).

Skill confidence also enables employees to approach JIN as a challenge rather than a threat. Employees who perceive themselves as capable are more likely to engage with skill-building opportunities and view organizational changes as growth opportunities rather than as destabilizing events (Shin et al., 2019; Tomas et al., 2019). This mindset reduces feelings of vulnerability and enhances adaptability, positioning SC as a critical mediator.

*H6*: Skill confidence mediates the relationship between (a) Co-Skilling participation and engagement, (b) Co-Skilling peer collaboration, (c) Co-Skilling learning effectiveness and job insecurity.

#### 2.3.7 Moderation of employee readiness for AI

The rapid integration of AI into workplace operations has intensified concerns about JIN. Many employees perceive AI-driven automation as a direct threat to job stability, particularly in roles with repetitive or routinized tasks that are highly automatable (Jiyoung and Jung, 2020; Koo et al., 2021). This apprehension often correlates with negative outcomes such as diminished mental health, reduced job satisfaction, disengagement, and higher turnover intentions (Di Stefano et al., 2020). However, the concept of ER for AI, encompassing an individual’s mindset, technical skills, and adaptability, offers a crucial mechanism for mitigating these effects (Liu and Zhan, 2020).

Core constructs such as POS, MEW, and SC play pivotal roles in shaping employees’ responses to JIN in AI-integrated environments. POS fosters a sense of reassurance by demonstrating an organization’s commitment to employee welfare, which mitigates stress and uncertainty (Beatriz et al., 2011; Xu et al., 2023). For example, an organization that provides clear communication about AI adoption and opportunities for upskilling creates a sense of trust, alleviating concerns about job loss. MEW, defined as emotional resilience and psychological stability, further enables employees to cope with the uncertainties of AI transitions (Gull et al., 2023; Sulaiman et al., 2020). Likewise, SC—rooted in self-efficacy—equips employees to perceive technological changes as opportunities for growth rather than as existential threats (Shin et al., 2019; Van Hootegem and De Witte, 2019).

Employee readiness for AI moderates the relationships between these factors and JIN. Individuals with high readiness for AI, characterized by an openness to learning, advanced technical skills, and adaptability, are better positioned to capitalize on POS, sustain MEW, and amplify their SC. For instance, employees in a technology company attending AI certification courses are more likely to view these programs as career advancement opportunities, reducing their job-related anxiety. High readiness enhances their capacity to translate organizational support into actionable strategies for personal development, thereby buffering against perceived insecurity (Liu and Zhan, 2020; Mathipriya et al., 2019).

Conversely, employees with low readiness for AI are more likely to experience heightened anxiety and vulnerability. These individuals may struggle to leverage POS effectively or maintain psychological resilience, exacerbating their sense of insecurity in AI-driven roles (Suseno et al., 2020). For example, retail employees unprepared for AI-driven inventory systems might feel overwhelmed, perceiving such technologies as insurmountable barriers rather than tools for operational efficiency. Targeted interventions—such as foundational AI training and programs designed to cultivate an AI growth mindset—can help bridge this readiness gap and mitigate adverse outcomes.

*H7*: Employee readiness for AI moderates the relationship between (a) perceived organizational support, (b) mental wellbeing, (c) skill confidence and job insecurity.

## 3 Methodology

### 3.1 Research design

This study employed a cross-sectional quantitative design targeting employees from industries such as manufacturing, healthcare, technology, banking, and retail. These industries were selected due to their substantial adoption of AI technologies, making them relevant for investigating Co-Skilling dynamics and workforce adaptation to AI (Mathipriya et al., 2019). The sampling frame was constructed using directories from the China AI Industry Alliance (AIIA), the Qichacha corporate registry, and participant lists from the World AI Conference (WAIC), all of which have been used in previous studies to ensure the comprehensiveness and relevance of sampling frames for organizational research (Cheng and Mugge, 2022; Zhang et al., 2021).

A total of 200 organizations were invited to participate in the study, with each organization requested to nominate at least seven employees meeting the inclusion criteria. These criteria required participants to (1) work in organizations actively using AI technologies and (2) have participated in Co-Skilling initiatives aimed at AI-related skill development. The criteria were informed by the need to ensure respondents’ relevance to the study’s focus on AI-enabled Co-Skilling (Koo et al., 2021). From the 200 organizations invited, 150 agreed to participate, yielding a total of 452 responses. After data cleaning to remove incomplete and ineligible responses, 437 valid responses were retained for analysis, reflecting a response rate of 96.7%, consistent with response rates in similar organizational studies (Hair et al., 2022).

To ensure proportional representation, a stratified random sampling method was adopted, which is well-established for enhancing representativeness in organizational research (Hair et al., 2022; Saunders et al., 2021). Organizations were categorized based on industry, size, and region. By industry, manufacturing contributed 32 organizations and 95 responses, healthcare contributed 29 organizations and 87 responses, technology contributed 42 organizations and 125 responses, banking contributed 23 organizations and 62 responses, and retail contributed 24 organizations and 68 responses. By organizational size, small organizations (<100 employees) accounted for 50 organizations and 134 responses, medium organizations (100–500 employees) accounted for 60 organizations and 181 responses, and large organizations (>500 employees) accounted for 40 organizations and 122 responses. By region, Beijing provided 45 organizations and 124 responses, Shanghai provided 40 organizations and 112 responses, Shenzhen provided 35 organizations and 108 responses, and Hangzhou provided 30 organizations and 93 responses. This stratification ensures the data reflects the diversity of organizations actively integrating AI technologies across China (Sun et al., 2020).

Organizations were contacted via email through their human resources or corporate communications departments. This method, widely recognized for its effectiveness in organizational studies (Saunders et al., 2021), was supplemented with follow-ups through phone calls and professional networks to enhance response rates and address potential participant concerns. Invitations outlined the study’s objectives, ensured confidentiality, and provided details about participation benefits, including access to anonymized data summaries upon study completion. Data collection was conducted over 3 weeks using a structured online questionnaire hosted on Tencent Forms, a platform widely used and trusted in China for academic and professional surveys. Screening questions embedded in the survey confirmed participant eligibility. This study followed the STROBE reporting guideline for cross-sectional observational research.

### 3.2 Measurement instrument

The measurement items of the constructs in this study were designed as reflective and adapted from previous high-impact academic studies, with slight modifications made to suit the research context (Table 1). After developing the questionnaire, it was pre-tested with eight respondents from diverse industries who were asked to review the items and provide feedback on their clarity and wording. Based on their comments, minor adjustments were made to improve the precision and comprehensibility of the items. To ensure the appropriateness and clarity of the questionnaire for a Chinese-speaking audience, the items were translated into Mandarin using a back-translation method. A five-point Likert scale, ranging from “strongly disagree” to “strongly agree,” was employed to measure all constructs, ensuring consistency and ease of interpretation.

**Table 1:** Demographics of the respondents.

Demographic variable	Category	Frequency	(%)
Industry	Manufacturing	95	21.7
	Healthcare	87	19.9
	Technology	125	28.6
	Banking	62	14.2
	Retail	68	15.6
Organizational size	Small (<100 employees)	134	30.7
	Medium (100–500 employees)	181	41.4
	Large (>500 employees)	122	27.9
Region	Beijing	124	28.4
	Shanghai	112	25.6
	Shenzhen	108	24.7
	Hangzhou	93	21.3
Gender	Male	248	56.8
	Female	189	43.2
Age group	18–29 years	105	24.0
	30–39 years	167	38.2
	40–49 years	125	28.6
	50 years and above	40	9.2
Education level	Bachelor’s Degree	265	60.6
	Master’s Degree	142	32.5
	Doctorate Degree	30	6.9
Job role	Technical/IT roles	157	35.9
	Operations/Production	103	23.6
	Administrative/HR	87	19.9
	Sales/Customer Service	53	12.1
	Managerial/Executive roles	37	8.5
Experience with AI tasks	Less than 1 year	92	21.0
	1–3 years	180	41.2
	More than 3 years	165	37.8
Monthly income (RMB)	Less than 10,000	98	22.4
	10,000–20,000	208	47.6
	Above 20,000	131	30.0

All constructs in this study were operationalized as reflective and were based on validated scales from prior research. The construct “ER” was measured using three items adapted from Mathipriya et al. (2019) and R. Liu and Zhan (2020), capturing employees’ willingness, confidence, and perceived opportunities related to AI adoption. The construct “JIN” was measured using three items adapted from De Witte (1999), focusing on perceived job stability in AI-integrated workplaces. The construct “LE” was assessed using four items adapted from Day et al. (2007), emphasizing the effectiveness of Co-Skilling initiatives in enhancing employees’ skills. “MEW” was measured with four items adapted Warwick-Edinburgh Mental Wellbeing Scale (WEMWBS), reflecting employees’ emotional resilience and psychological health. The construct “PC” included five items adapted from Van Der Vegt et al. (2006), highlighting knowledge exchange and support among peers. “PE” was measured using five items adapted from Spreitzer (1995), focusing on employees’ autonomy and confidence in decision-making. The construct “POS” included four items adapted from Eisenberger and Stinglhamber (2011), capturing organizational care, recognition, and investment in employees. Lastly, “SC” was assessed using three items adapted from Chen et al. (2014), reflecting employees’ self-efficacy in skill acquisition and application.

Demographic information, such as age, gender, industry type, and organizational size, was also collected for further analysis. The descriptive statistics for these variables were analyzed using SPSS version 26, providing insights into the sample characteristics. To assess the questionnaire’s reliability and validity, the 30 pilot responses were analyzed using Cronbach’s alpha for internal consistency and exploratory factor analysis for construct validity. The questionnaire’s validity and reliability were tested using Partial Least Squares Structural Equation Modeling (PLS-SEM).

## 4 Data analysis

### 4.1 Participants

The demographic profile of respondents in this study reflects a diverse and representative sample across industries, organizational sizes, and regions within China. Notably, the majority of participants were from technology (28.6%), manufacturing (21.7%), and healthcare (19.9%) sectors, consistent with the study’s focus on industries adopting AI technologies. Organizational size was well-distributed, with medium-sized enterprises (41.4%) being the most represented, followed by small (30.7%) and large organizations (27.9%). Regionally, responses were fairly balanced among major cities, including Beijing (28.4%), Shanghai (25.6%), Shenzhen (24.7%), and Hangzhou (21.3%), reflecting key economic hubs with substantial AI activity.

Gender distribution leaned male (56.8%), with females comprising 43.2%, aligning with typical workforce compositions in technology-intensive industries. The age group 30–39 years dominated (38.2%), followed by 40–49 years (28.6%) and younger participants aged 18–29 years (24.0%), suggesting a workforce predominantly in early to mid-career stages. Educational attainment was high, with 60.6% holding bachelor’s degrees and 32.5% master’s degrees, indicating a skilled workforce suitable for AI adoption and Co-Skilling initiatives.

Job roles were diverse, with technical/IT roles (35.9%) and operations/production (23.6%) being prominent, reflecting the technical orientation of AI-driven tasks. Experience with AI was balanced, with most participants having 1–3 years (41.2%) or more than 3 years (37.8%), indicating significant exposure to AI in workplace settings. Monthly income showed moderate variability, with nearly half earning between 10,000–20,000 RMB (47.6%), while 30.0% earned above 20,000 RMB, reflecting the economic viability of participants in AI-intensive roles.

**Figure 2:**
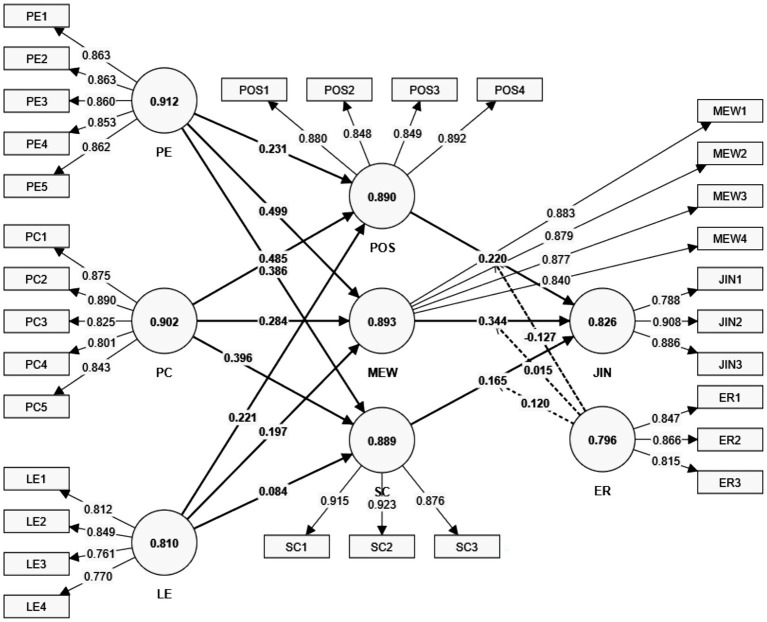
Measurement model.

This demographic analysis underscores the study’s robust design and its alignment with the target population of employees engaged in AI-related roles across diverse organizational contexts in China (Figure 2).

### 4.2 Measurement model statistics

The evaluation of the measurement model confirms the robustness and validity of the constructs used in this study. Key indicators, including outer loadings, variance inflation factors (VIF), Cronbach’s alpha, composite reliability (CR), and average variance extracted (AVE), validate the reliability, internal consistency, and discriminant validity of the constructs. These findings provide a solid foundation for interpreting the relationships among Co-Skilling dimensions, JIN, and employee outcomes in AI-driven organizational contexts (Table 2).

**Table 2:** Measurement model statistics.

Construct	Items	OL	VIF	CA	CR	AVE
ER	ER1	0.847	1.716	0.796	0.880	0.711
	ER2	0.866	1.874			
	ER3	0.815	1.579			
JIN	JIN1	0.788	1.518	0.826	0.897	0.744
	JIN2	0.908	2.612			
	JIN3	0.886	2.377			
LE	LE1	0.812	1.733	0.810	0.876	0.638
	LE2	0.849	2.051			
	LE3	0.761	1.547			
	LE4	0.770	1.668			
MEW	MEW1	0.883	2.554	0.893	0.926	0.757
	MEW2	0.879	2.599			
	MEW3	0.877	2.513			
	MEW4	0.840	2.177			
PC	PC1	0.875	2.895	0.902	0.927	0.718
	PC2	0.890	3.174			
	PC3	0.825	2.186			
	PC4	0.801	2.072			
	PC5	0.843	2.261			
PE	PE1	0.863	3.053	0.912	0.934	0.740
	PE2	0.863	3.048			
	PE3	0.860	2.587			
	PE4	0.853	2.491			
	PE5	0.862	2.628			
POS	POS1	0.880	2.610	0.890	0.924	0.753
	POS2	0.848	2.140			
	POS3	0.849	2.253			
	POS4	0.892	2.864			
SC	SC1	0.915	2.972	0.889	0.931	0.819
	SC2	0.923	3.156			
	SC3	0.876	2.163			

All items demonstrate strong indicator reliability, with outer loadings exceeding the threshold of 0.70 (Hair et al., 2022). Cronbach’s alpha values range from 0.796 to 0.912, and CR exceeds the critical value of 0.70 for all constructs, indicating high internal consistency. These metrics confirm that the scales reliably measure the latent constructs. Furthermore, the AVE values for all constructs surpass 0.50, ensuring convergent validity by demonstrating that more than 50% of the variance in the items is attributable to their respective constructs (Fornell and Larcker, 1981).

The VIF values are well below the threshold of 5, suggesting no multicollinearity concerns among the items (Diamantopoulos and Siguaw, 2006). This supports the stability and reliability of the regression estimates within the structural model. Discriminant validity is supported by both the heterotrait-monotrait (HTMT) ratio and the Fornell-Larcker criterion. The HTMT ratios between constructs remain below the recommended threshold of 0.85, affirming that the constructs are distinct (Henseler, 2017). Additionally, the Fornell-Larcker criterion reveals that each construct’s AVE exceeds its squared correlation with any other construct, further validating discriminant validity (Table 3).

**Table 3:** Discriminant validity.

Construct	ER	JIN	LE	MEW	PC	PE	POS	SC	ER x SC	ER x POS	ER x MEW
HTMT											
ER											
JIN	0.801										
LE	0.760	0.746									
MEW	0.831	0.895	0.791								
PC	0.829	0.836	0.822	0.822							
PE	0.812	0.846	0.644	0.840	0.698						
POS	0.849	0.844	0.811	0.859	0.822	0.732					
SC	0.841	0.823	0.681	0.795	0.780	0.760	0.838				
ER × SC	0.474	0.391	0.302	0.455	0.370	0.422	0.414	0.475			
ER × POS	0.449	0.436	0.272	0.419	0.334	0.358	0.424	0.418	0.832		
ER × MEW	0.460	0.420	0.281	0.500	0.346	0.446	0.389	0.426	0.840	0.847	
FLC											
ER	0.843										
JIN	0.729	0.862									
LE	0.611	0.613	0.799								
MEW	0.748	0.772	0.676	0.870							
PC	0.705	0.726	0.705	0.743	0.848						
PE	0.693	0.732	0.559	0.791	0.643	0.860					
POS	0.716	0.753	0.692	0.768	0.789	0.666	0.868				
SC	0.708	0.706	0.579	0.712	0.704	0.687	0.747	0.905			

Within the study’s context, these results underscore the robustness of the constructs related to ER, SC, MEW, POS, and their interactions. The strong reliability and validity of these constructs provide confidence in exploring their relationships with Co-Skilling dimensions and JIN. For instance, the constructs related to AI readiness and organizational support exhibit strong validity, supporting their theoretical role in buffering JIN and enhancing employee outcomes. Interaction terms such as ER x SC and ER x MEW meet discriminant validity requirements, validating their use in examining moderating effects within the model.

### 4.3 Model fit and predictive relevance

The model fit and predictive relevance were assessed using established metrics such as *R*^2^, adjusted *R*^2^, *Q*^2^predict, RMSE, and MAE, as recommended in structural equation modeling literature (Sarstedt et al., 2022). The *R*^2^ values reflect the explanatory capacity of the model, with JIN (*R*^2^ = 0.700), MEW (*R*^2^ = 0.738), POS (*R*^2^ = 0.689), and SC (*R*^2^ = 0.593) demonstrating substantial explained variance. The adjusted *R*^2^values closely aligned with R^2^, confirming the model’s robustness and mitigating concerns of overfitting (Henseler, 2017) (Table 4).

**Table 4:** Model fit and predictive statistics.

Construct	*R* ^2^	*R*^2^ adjusted	*Q*^2^ predict	RMSE	MAE
JIN	0.700	0.695	0.665	0.582	0.421
MEW	0.738	0.736	0.729	0.524	0.368
POS	0.689	0.687	0.679	0.570	0.393
SC	0.593	0.590	0.582	0.652	0.458

Predictive relevance, indicated by *Q*^2^ predict values, was strong across constructs (*Q*^2^ > 0), with MEW (*Q*^2^ = 0.729) exhibiting the highest predictive accuracy. These values exceed the recommended threshold, validating the model’s predictive power (Chin, 1998; Geisser, 1974). Error metrics, such as RMSE and MAE, corroborate the model’s precision, with MEW demonstrating the lowest residual variance (RMSE = 0.524, MAE = 0.368). These low error values highlight the model’s reliability and validity in capturing relationships among constructs (Hair et al., 2022).

### 4.4 NCA statistics

The Necessary Condition Analysis (NCA) results presented in Table 5 provide insights into the bottlenecks influencing the dependent constructs of the model, including JIN, ER, LE, MEW, PC, PE, POS, and SC. The bottleneck table reports condition levels required to achieve specific outcome levels using the CR-FDH approach.

**Table 5:** Bottleneck tables – CR-FDH.

Construct	JIN	ER	LE	MEW	PC	PE	POS	SC
00%	−2.995	0.000	0.000	0.000	0.000	0.000	0.000	0.000
10%	−2.562	0.000	0.000	0.000	0.000	0.000	0.000	0.000
20%	−2.130	0.000	0.000	0.000	0.000	0.000	0.000	0.000
30%	−1.698	0.000	0.000	0.000	0.000	1.781	0.000	0.000
40%	−1.265	0.000	0.000	0.000	0.000	2.036	0.000	0.000
50%	−0.833	0.000	0.000	0.000	0.000	3.053	0.000	0.000
60%	−0.400	0.000	0.000	2.290	0.000	3.562	0.000	0.000
70%	0.032	0.000	0.000	2.799	3.562	6.870	0.000	0.000
80%	0.464	2.545	3.308	5.344	8.142	13.486	5.089	3.817
90%	0.897	17.048	4.326	10.178	24.682	29.008	9.924	4.580
100%	1.329	78.117	7.125	19.847	52.926	66.412	30.025	4.580

The findings reveal that the conditions vary significantly across constructs and outcome levels. For instance, at the 80% outcome level, SC requires 3.817 units of ER and 5.089 units of POS, highlighting these constructs’ foundational role in enhancing SC. Similarly, PE emerges as a critical requirement at higher outcome levels, with increasing values across all stages, reflecting its indispensable influence on MEW and JIN.

At the 90 and 100% levels, the dependency on constructs such as PC and PE intensifies, with PC reaching a bottleneck value of 24.682 at 90% and 52.926 at 100%. This indicates the necessity of substantial competence for achieving high levels of MEW, SC, and other critical outcomes. These results are consistent with prior studies emphasizing the role of psychological resources in mitigating JIN and fostering employee resilience (Henseler, 2017).

**Figure 3:**
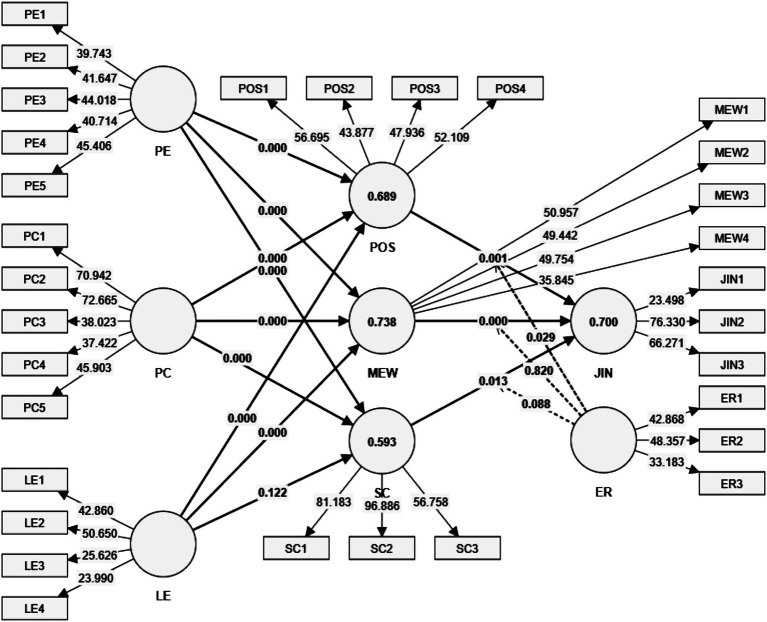
Structural model.

Overall, the NCA results demonstrate the incremental and conditional importance of specific predictors, guiding organizations in prioritizing interventions such as fostering psychological empowerment and enhancing perceived competence to achieve desired workforce outcomes in AI-integrated workplaces (Figure 3).

### 4.5 Hypothesis testing and discussion

The findings from the structural model analysis and the Necessary Conditions Analysis (NCA) reveal important insights into the interplay of PE, PC, LE, and mediators like POS, MEW, and SC in addressing JIN in AI-driven workplaces. The results offer significant theoretical and practical implications, as summarized in Table 6.

**Table 6:** Structural model analysis and Necessary conditions analysis.

Hypothesis Path		SEM				Construct	NCA			Support
		Ori Sam	T stat	P val	f^2^		Ori Eff	95.0%	Per p val	
H1A	PE - > POS	0.231	4.568	0.000	0.097	ER	0.098	0.021	0.000	Yes
H1B	PE - > MEW	0.499	8.769	0.000	0.537	LE	0.022	0.006	0.001	Yes
H1C	PE - > SC	0.386	6.272	0.000	0.206	MEW	0.126	0.026	0.000	Yes
H2A	PC - > POS	0.485	6.867	0.000	0.312	PC	0.102	0.008	0.000	Yes
H2B	PC - > MEW	0.284	4.101	0.000	0.127	PE	0.273	0.033	0.000	Yes
H2C	PC - > SC	0.396	5.538	0.000	0.159	POS	0.070	0.005	0.000	Yes
H3A	LE - > POS	0.221	4.088	0.000	0.076	SC	0.020	0.007	0.010	Yes
H3B	LE - > MEW	0.197	4.536	0.000	0.072					Yes
H3C	LE - > SC	0.084	1.548	0.122	0.008					No
H4A	PE - > POS - > JIN	0.051	2.685	0.007						Yes
H4B	PC - > POS - > JIN	0.106	3.008	0.003						Yes
H4C	LE - > POS - > JIN	0.049	2.616	0.009						Yes
H5A	PE - > MEW - > JIN	0.171	4.583	0.000						Yes
H5B	PC - > MEW - > JIN	0.097	3.118	0.002						Yes
H5C	LE - > MEW - > JIN	0.068	3.522	0.000						Yes
H6A	PE - > SC - > JIN	0.063	2.315	0.021						Yes
H6B	PC - > SC - > JIN	0.065	2.163	0.031						Yes
H6C	LE - > SC - > JIN	0.014	1.166	0.244						No
H7A	ER x POS - > JIN	−0.127	2.189	0.029	0.027					Yes
H7B	ER x MEW - > JIN	0.015	0.227	0.820	0.000					No
H7C	ER x SC - > JIN	0.120	1.707	0.088	0.023					No

In alignment with resource-based and empowerment theories (Alkasim and Prahara, 2020; Eisenberger and Stinglhamber, 2011), the analysis demonstrates how PE, PC, and LE jointly mitigate JIN by shaping POS, MEW, and SC. The particularly strong paths from PE to these mediators underscore that employees who feel empowered also perceive greater organizational care (*β* = 0.231, *p* < 0.001), experience improved emotional resilience (*β* = 0.499, *p* < 0.001), and report higher levels of skill-related self-assurance (*β* = 0.386, *p* < 0.001). These results accord with studies suggesting that autonomy, meaningful participation, and ownership of tasks facilitate both psychological wellbeing and stronger commitment to shared goals (Dunne et al., 2021; Perkins et al., 2020). The equally robust influence of PC on POS (*β* = 0.485, *p* < 0.001), MEW (*β* = 0.284, *p* < 0.001), and SC (*β* = 0.396, *p* < 0.001) echoes findings that competence-building programs and collaborative problem-solving not only help employees gain mastery over tasks but also assure them of the organization’s investment in their development (Hayton et al., 2011; Simosi, 2012).

Meanwhile, LE strengthens POS and MEW (both *p* < 0.001), which reinforces perspectives in adult learning literature that effective development opportunities confer a sense of institutional backing and enhance psychological wellbeing (Billett, 2001). However, the non-significant path from LE to SC (*β* = 0.084, *p* = 0.122) points to an ongoing debate on whether classroom or formal learning alone suffices to instill genuine confidence in complex tasks (Day et al., 2007). Several researchers suggest that repeated practice, iterative feedback, and peer validation play a more direct role in building one’s belief in their proficiency (Billett, 2001; Norman and Hyland, 2003), especially for those with limited prior experience or low self-efficacy.

The mediation analyses further support the view that POS, MEW, and SC serve as essential channels through which PE, PC, and LE lower JIN. For instance, POS significantly mediates the impact of PC on insecurity (*β* = 0.106, *p* < 0.01), illustrating that skill enhancement convinces employees of organizational commitment, which in turn buffers fears about AI-driven changes (dos Santos et al., 2022; Xu et al., 2023). MEW, likewise, acts as a psychological stabilizer (e.g., PE → MEW → JIN, *β* = 0.171, *p* < 0.001), aligning with evidence that structured emotional support—be it through peer-led coaching or social learning forums—helps employees cope with transformation-related stress (Briand et al., 2023; Kundi et al., 2020). SC emerges as a partial conduit, mediating the connection between PE (*β* = 0.063, *p* < 0.05) and PC (*β* = 0.065, *p* < 0.05) with JIN, confirming earlier work that self-assuredness in performing AI-related tasks translates into heightened resilience (Day et al., 2007). However, the lack of a significant mediation link between LE and JIN through SC (*β* = 0.014, *p* = 0.244) further underscores that SC is not automatically imparted by formal learning programs; rather, it demands experiences where learners apply and validate newly acquired knowledge (Billett, 2001).

Employee readiness for AI significantly moderates the relationship between POS and JIN (*β* = −0.127, *p* < 0.05), suggesting that those already comfortable with AI-based processes respond more favorably to supportive organizational signals (Mathipriya et al., 2019). By contrast, ER does not strongly influence how MEW (*β* = 0.015, *p* = 0.820) or SC (*β* = 0.120, *p* = 0.088) affect JIN, mirroring research that positions emotional wellbeing and underlying SC as relatively stable buffers independent of one’s readiness for new technologies (Shin et al., 2019).

Finally, the NCA pinpoints PE, SC, MEW, and PC as critical thresholds for reducing JIN in AI-intensive environments. The evidence that high SC is needed to achieve substantial (e.g., ~80%) reductions in insecurity resonates with prior claims that iterative, hands-on training fosters both mastery and psychological safety (Norman and Hyland, 2003). MEW emerges as similarly pivotal at higher thresholds, consistent with findings linking emotional wellbeing to lower stress and reduced vulnerability during organizational shifts (Haar and Brougham, 2011). In earlier stages of adaptation, PE and PC appear vital for jumpstarting confidence and commitment, with activities such as collaborative AI workshops or open dialogue on impending changes increasing employees’ sense of control (Suseno et al., 2020). Taken together, these results illustrate a nuanced interplay of psychological and organizational factors underpinning how workers navigate AI-driven transformations while maintaining a sense of security and wellbeing.

## 5 Implications of this study

### 5.1 Theoretical implications

This research expands frameworks such as SLR (Bandura, 1977) and the JD-R Model (Bakker and Demerouti, 2014) by highlighting how Co-Skilling dimensions—involving participation, peer collaboration, and LE—address the unique challenges of AI-driven workplaces. Rooted in SLR, the findings confirm that individuals acquire skills, behaviors, and attitudes by observing, imitating, and modeling in social contexts. Specifically, the study shows how collaborative environments enable employees to learn from peers and mentors, thereby enhancing SC and MEW. The significant positive effects of peer collaboration on SC and MEW underscore the power of vicarious learning and social reinforcement in cultivating self-efficacy (Curtis et al., 2016).

Employee readiness for AI emerges as an important extension of SLR, moderating the relationship between POS and JIN. This finding highlights how adaptability and technological competence shape the effectiveness of social learning mechanisms in reducing insecurity (R. Liu and Zhan, 2020; Mathipriya et al., 2019). By recognizing the influence of AI readiness on learning outcomes, the study broadens Social Learning Theory to account for technological shifts that require employees to build new skills even as they cope with evolving work processes.

In incorporating Co-Skilling dimensions as crucial resources, the study also advances the JD-R Model, which holds that job resources can buffer the impact of demands on employee wellbeing and performance. Here, POS, MEW, and SC serve as mediators linking Co-Skilling initiatives to reduced JIN. For instance, POS significantly mediates the relationship between Perceived Empowerment (PE) and JIN, reflecting how organizational care and support can lessen anxiety tied to AI adoption (Sulaiman et al., 2020; Xu et al., 2023). The role of ER further highlights the conditional nature of these resources in AI-focused settings: employees with higher readiness capitalize more effectively on POS, while the nonsignificant moderating effects of ER on MEW and SC suggest that psychological and skill-based resources may need more fine-tuned interventions to align with individual readiness (Suseno et al., 2020).

Additionally, the NCA reveals that higher thresholds of SC and MEW require robust investments in empowerment and skill-building, aligning with calls for a phased resource-allocation approach (Leonard and Marquardt, 2010; Maurer, 2001). These insights refine the JD-R Model by emphasizing the importance of supporting employees at multiple stages: building foundational resources such as empowerment and POS before scaling up to comprehensive skill-confidence programs.

### 5.2 Practical implications

Organizations navigating AI-related changes can draw from these findings to mitigate JIN and bolster workforce adaptability. A central recommendation is to implement Co-Skilling initiatives that strategically blend collaborative training with problem-solving exercises tailored to specific technologies. For example, structured AI workshops or cross-functional “innovation sprints” can foster peer learning and skill application, while pairing employees with varied AI expertise promotes knowledge sharing and collective growth.

Given the central role of POS in mediating Co-Skilling outcomes, organizations should visibly demonstrate their commitment to employee development. Transparent communication about AI integration—along with readily available upskilling opportunities—can ease concerns about job displacement. Incorporating interactive digital platforms with real-time feedback mechanisms can make organizational support more tangible and align individual progress with broader strategic goals.

The positive impact of MEW highlights the need for psychological safety and emotional resilience in technology-driven environments. Integrating mental health services or mindfulness workshops into Co-Skilling programs normalizes wellbeing as part of organizational culture. AI-based wellness tools, such as chatbots offering personalized support, can seamlessly embed mental health assistance into daily workflows.

In parallel, SC underscores the value of hands-on experience in cultivating self-efficacy. AI simulation exercises, hackathons, and mentorship programs—where feedback is both constructive and frequent—provide safe contexts for practice, helping employees internalize newly acquired skills. By making SC-building activities a core part of organizational learning, firms can strengthen employees’ ability to manage emerging challenges with confidence.

The moderation effect of ER suggests that interventions should be tailored to varying degrees of AI readiness. Employees with lower readiness may benefit most from fundamental AI literacy programs that utilize gamified learning or intuitive demonstration tools, while those with higher readiness can assume leadership roles in AI projects or pursue advanced certifications. Segmenting training in this way ensures that each individual’s capacity and comfort level is appropriately addressed.

Insights from the NCA further indicate that significant reductions in JIN hinge on achieving particular thresholds of empowerment, skill-building, and wellbeing support. Organizations should therefore develop staged resource strategies, first ensuring adequate empowerment and support mechanisms are in place before expanding Co-Skilling across the workforce.

### 5.3 Limitations and future research

First, this study relies on a cross-sectional design conducted in a rapidly evolving AI context. While this approach is appropriate for capturing current perceptions of co-skilling, perceived organizational support, mental wellbeing, and job insecurity, it does not allow us to observe how these relationships unfold over time as AI technologies diffuse and organizational practices mature. Future research could adopt longitudinal designs to track dynamic shifts in job insecurity and psychological resources as employees repeatedly participate in co-skilling initiatives.

Second, our quantitative survey approach limits our ability to capture the rich, contextualized experiences of employees who are directly engaged in co-skilling activities. Follow-up qualitative studies—such as interviews, focus groups, or ethnographic observations—could illuminate the specific challenges, tensions, and coping strategies that employees use when navigating AI-driven change. A multi-stakeholder design that includes both employees and employers/HR leaders would be particularly valuable for understanding the demand–supply dynamics of co-skilling: how organizations design and resource such programs, and how employees perceive, access, and benefit from them.

Third, our empirical evidence is drawn from organizations in China, and the institutional and cultural context may shape both the design of co-skilling and employees’ reactions to AI-induced job insecurity. Cross-country comparative studies could examine whether similar co-skilling mechanisms and psychological resources operate in different socio-cultural or regulatory environments (for example, comparing collectivist versus individualist cultures or advanced versus emerging economies). Such work would substantially enhance the global generalizability and practical relevance of co-skilling as a strategy for managing AI-driven workforce transformation.

## 6 Conclusion

This study illuminates how Co-Skilling initiatives can mitigate JIN in AI-enabled workplaces by leveraging organizational support, MEW, SC, and AI readiness. The significant relationships among participation, peer collaboration, and LE validate that Co-Skilling enhances both psychological and skill-based resources, thereby reducing JIN. Mediation tests reveal that POS, MEW, and SC operate as key conduits through which Co-Skilling practices influence JIN, while moderation tests indicate that ER conditions the impact of POS on insecurity. However, certain nonsignificant effects, especially those linked to SC and ER, signal that other contextual factors may shape how employees benefit from Co-Skilling in AI-centric settings. By demonstrating how Social Learning Theory and the JD-R Model apply to AI-driven organizational contexts, the study underscores the multifaceted nature of workforce adaptation.

## Data Availability

The raw data supporting the conclusions of this article will be made available by the authors, without undue reservation.
